# *Klebsiella pneumoniae*-related brain abscess and meningitis in adults: Case report

**DOI:** 10.1097/MD.0000000000028415

**Published:** 2022-01-14

**Authors:** Jingru Zhao, Tiantian Huo, Xintong Luo, Fan Lu, Shuo Hui, Baoming Yang

**Affiliations:** a Department of Neurology, Hebei General Hospital, 348th Heping West Road, Shijiazhuang, P.R. China; b Department of Hepatobiliary Surgery, the Fourth Hospital of Hebei Medical University, 12th Jiankang Road, Shijiazhuang, P.R. China.

**Keywords:** brain abscess, *Klebsiella*, meningitis

## Abstract

**Introduction:**

*Klebsiella pneumoniae* is once thought to be a less common cause of brain abscess in adults and is mainly hospital-acquired. Community-acquired CNS infection (brain abscess and meningitis) caused by *K pneumoniae* without other metastatic septic abscesses is exceedingly rare. Therefore, we present a rare adult patient with invasive cerebral abscess and meningitis without other invasive abscesses related to *K pneumoniae*.

**Patient concerns:**

A 64-year-old woman experienced a sudden onset of severe continuous headache accompanied by intermittent nausea, vomiting, and fever. Meanwhile, she experienced tinnitus and had a feeling of swelling in the right ear.

**Diagnosis:**

Cranial magnetic resonance imaging revealed abnormal hyperintensity signals in the left head of the caudate nucleus. The next generation sequencing of cerebral spinal fluid showed infection with *K pneumoniae*. The patient was diagnosed with *K pneumoniae*-related brain abscesses and meningitis.

**Interventions:**

Antibacterial treatment was carried out for 2 months.

**Outcomes:**

The patient recovered well.

**Conclusion:**

Despite the progress of modern neurosurgical techniques, new antibiotics, and modern imaging techniques, brain abscesses are still a potentially fatal infection. Streptococci are common organisms that result in brain abscesses. Nevertheless, *Klebsiella* species, once thought to be a less common cause of brain abscess in adults, has become an increasingly important cause of brain abscess, especially in Asia.

## 1 Introduction

Despite the progress of modern neurosurgical techniques, new antibiotics, and modern imaging techniques, brain abscess is still a potentially fatal infection caused primarily by spread from infected parameningeal or remote foci.^[1]^ Streptococci are common organisms that result in brain abscesses. Nevertheless, *Klebsiella* species, once thought to be a less common cause of brain abscess in adults, has become an increasingly important cause of brain abscesses in Asia.^[2]^*Klebsiella pneumoniae* is mainly hospital-acquired.^[3]^ Community-acquired CNS infection (brain abscess and meningitis) caused by *K pneumoniae* without other metastatic septic abscesses is exceedingly rare. Therefore, we present a rare adult patient with invasive cerebral abscess and meningitis without other invasive abscesses related to *K pneumoniae*.

## 2 Case presentation

A 64-year-old woman experienced sudden onset severe continuous headache, especially in the front temporal occipitoparietal areas, accompanied by intermittent nausea and vomiting on June 21, 2020. Meanwhile, she felt tinnitus and had a feeling of swelling in the right ear, but without fever, lateral limb weakness, blurred vision, and loss of consciousness. Electroencephalogram examination revealed mild abnormalities. Upon admission on June 26, the patient developed a fever with the highest temperature reaching 39.5°C. Neurological examination revealed obvious nuchal rigidity with 4 transverse fingers under the chin. However, he had an intact sensation and full limb power with a negative bilateral Babinski sign.

**Figure 1:**
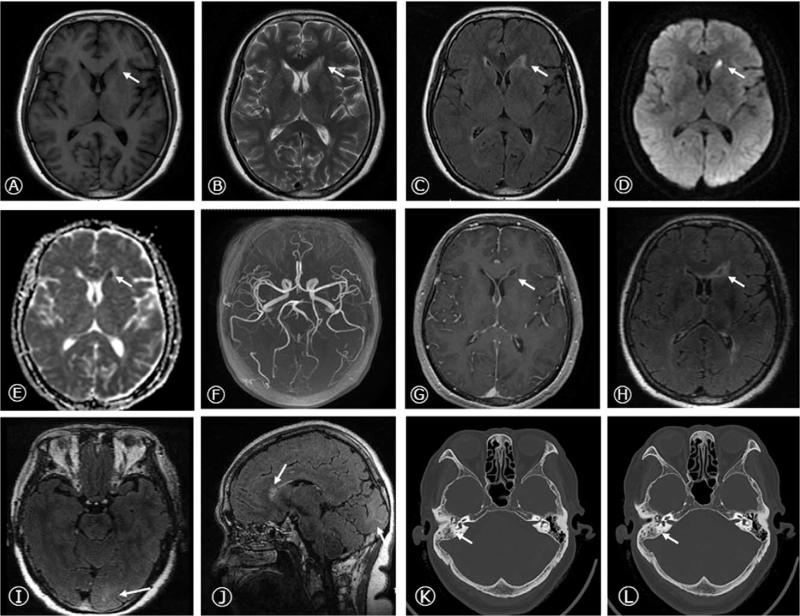
Brain MRI and Temporal bone CT at admission. Axial T1-weighted sequence, T2 -weighted sequence, FLAIR sequence (A-C), DWI (D), and ADC (E) sequences were imaged and showed abnormal signals in the left head of caudate nucleus, next to the anterior horn of lateral ventricle. No significant abnormality was observed in the cerebral MRA (F). Cranial SPGR and meningeal CUBE enhancement displayed the ring-enhancement in the left head of caudate nucleus and meningeal linear enhancement in left occipital lobe on axial (G-I) and sagittal images (J). The temporal bone CT showed the right otitis mastoidea (K, L). CT = computed tomography, MRI = magnetic resonance imaging.

Cranial magnetic resonance imaging (MRI) revealed abnormal hyperintensity signals in the left head of the caudate nucleus, with T1-weighted sequence of hypointensity, T2-weighted sequence of hyperintensity, FLAIR sequence of slightly hyperintensity, and diffusion restriction on diffusion-weighted imaging (DWI) and apparent diffusion coefficient (ADC) at the corresponding position. No significant abnormalities were observed on cerebral magnetic resonance angiography (MRA). Therefore, we further conducted cranial SPGR and meningeal CUBE enhancement, which displayed ring enhancement with consistent thickness next to the anterior horn of the left lateral ventricle and meningeal linear enhancement in the left occipital lobe, suggesting a high probability of infectious diseases of brain abscess and meningitis (Fig. 1A-J).

Blood analysis showed elevated levels of neutrophil granulocyte percentage (84.6%), C-reactive protein (CRP) (238.88 mg/L), erythrocyte sedimentation rate (ESR; 82 mm/h), and procalcitonin (0.228 ng/mL). Accordingly, we gave the patient anti-infective therapy of ceftriaxone (2 g Q12 hour) and metronidazole (0.5 g Q8 hour) by experience. The temperature began to decrease, fluctuating between 37.0°C and 37.8°C. The antinuclear antibody, hepatitis, and tumor screening, coagulation test, thyroid function, vasculitis screening, Brucella agglutination test, tiger red plate agglutination test, Brucella IgG antibody detection, Widal and Weil-Felix reaction, anti-human globulin test, T cell test for tuberculous infection, HIV, TP, blood culture, cardiac ultrasound, chest computed tomography (CT), cranial CT, and abdominal CT showed no obvious abnormalities. The CT scan of the temporal bone and paranasal sinuses showed right otitis mastoidea (Fig. 1K, L). The otolaryngology consultation considered the right otitis media, and 0.2 mL of sallow exudate was extracted.

**Figure 2:**
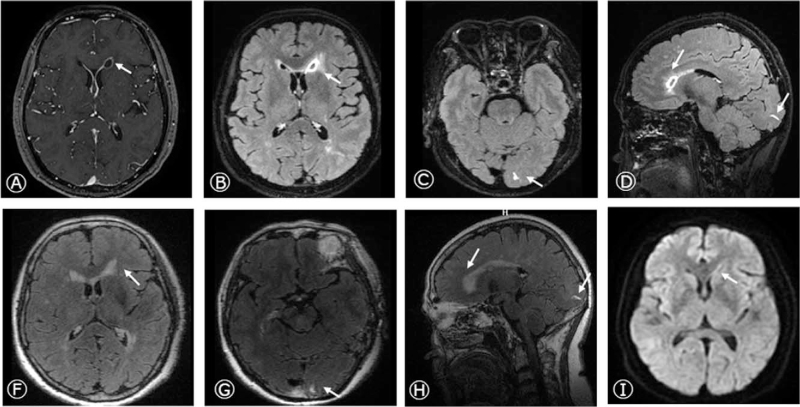
CSF cytology results on June 29 (A), July 7 (B), July 22 (C), and August 31 (D), respectively. CSF = cerebral spinal fluid.

**Figure 3:**
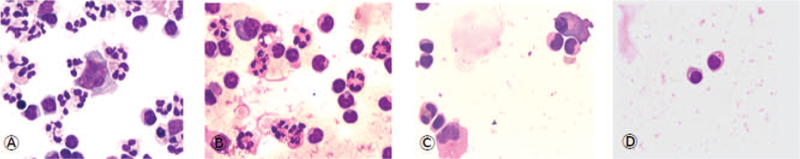
Brain MRI after therapy. Cranial SPGR (A) and meningeal CUBE enhancement (B-D) displayed the ring-enhancement in the left head of caudate nucleus and linear enhancement in left occipital lobe on axial and sagittal images on July 10, 2020. The cranial SPGR (E), meningeal CUBE enhancement (F-H), DWI (I), and ADC (J) revealed nearly disappeared ring and linear enhancement on August 31, 2020. MRI = magnetic resonance imaging.

The common cerebral spinal fluid (CSF) results on June 29 were shown in Table 1 and Figure 2. CSF cultures, acid-fast dyeing, and India ink staining were negative. The next generation sequencing (NGS) of CSF showed infection with *K pneumoniae*. Ceftriaxone and metronidazole were replaced by meropenem to broaden the antimicrobial spectrum, and 20% mannitol (125 mL Q8 hour) was added to reduce the intracranial pressure. The patient's body temperature dropped below 37.3°C, and the symptoms of headache and fever were alleviated. The CSF results improved on July 6 (Table 1, Fig. 2). The patient was asked to discharge. The cranial SPGR and meningeal CUBE enhancement on July 10 displayed the abscess cavity and wall were larger and thicker than before (Fig. 3A-D). The patient returned to the local hospital and received ceftriaxone injections for 8 weeks. The CSF results improved on July 22 (Table 1, Fig. 2). Almost 2 months after the onset (August 31), the CSF results returned nearly normal (Table 1, Fig. 2), and the lesions on MRI nearly disappeared (Fig. 3E-J). She did not complain of any uncomfortable until December 28. The study protocol was approved by the Ethics Committee of the Hebei General Hospital, and the patient provided consent for publication of this case report.

**Table 1:** CSF results.

CSF data	2020-6-29	2020-7-6	2020-7-22	2020-8-31
CSF appearance	Yellow	Light yellow	Transparent	Transparent
CSF pressure (mmH_2_O)	200	160	150	130
Protein (mg/dL)	74.49	73.98	31.43	28.65
Glucose 1(mg/dL)	70.92	45.98	51.98	64.84
Chlorine (mmol/L)	120	117	128	129
Leukocyte count (×10^6^/L)	400	232	57	6
Percentage of neutrophils (%)	59	30	1	1
Percentage of lymphocyte (%)	30	61	84	95

CSF = cerebral spinal fluid.

## 3 Discussion

Purulent meningitis is often associated with purulent encephalitis or brain abscesses. Through further MRI imaging, CSF analysis, NGS results, and therapeutic effects, we confirmed that this patient had a *K pneumoniae*-associated solitary brain abscess secondary to meningitis, which was relatively rare in CNS infection in adults without a history of trauma, surgery, or cerebrospinal fluid leakage.

*K pneumoniae*, a gram-negative Enterobacteriaceae, is mainly hospital-acquired and tends to result in multiple abscesses in patients with a fragile immune system.^[4]^ Nevertheless, community-acquired *K pneumoniae*-related solitary brain abscesses without other metastatic septic abscesses are rare. The patient in our case seldom visited the hospital before being hospitalized this time, and without other medical history except chronic otitis media, suggesting a high probability of community-acquired *K pneumoniae* focal CNS without invasion to other organs, which is very rare and is in accordance with a previous report.^[5]^ Accordingly, it gave us a reminder that *K pneumoniae* could not be completely excluded in CNS community-acquired infections, especially in Asia. NGS is still necessary to guide the diagnosis and treatment of CNS infectious diseases.

Brain abscesses usually result from predisposing factors, such as otitis media, mastoiditis, trauma, post-neurosurgery, human immunodeficiency virus (HIV), and long-term treatment with immunosuppressive drugs. Brain abscesses from chronic otitis media and mastoiditis are usually caused by *Streptococcus* species and account for about half of the cases. However, it is notable that Enterobacteriaceae have not been ignored in recent years. In this patient, based on the history, the sense of ear swelling, temporal bone CT, excretion from the ear, negative blood culture, and solitary brain abscess without multiple invasions, we speculated that the infectious sources might be related to the middle ear and mastoids. Nevertheless, it was a pity that we had not obtained the bacterial culture of the yellow exudate exacted from the ear, which might be a possible way to prove the brain abscess sources. Accordingly, we should try the best to perfect relevant inspection to specify the pathogenesis in future clinical work.

Empirical anti-infective treatment for patients with contiguous spread from a parameningeal infection and no history of neurosurgery consists of ceftriaxone/cefotaxime combined with metronidazole or meropenem. Cefotaxime or ceftriaxone is recommended for treatment based on isolated pathogens of Enterobacteriaceae. The duration of intravenous antimicrobial therapy in patients with bacterial brain abscesses has traditionally been 6 to 8 weeks.^[6]^ Important criteria for evaluating treatment are the neurologic condition of the patient and abscess size on image examination. In this patient, ceftriaxone combined with metronidazole and meropenem were all used to cover anaerobes and broaden the antibacterial spectrum in the early stage, and ceftriaxone used for nearly 2 months had good therapeutic effects.

In summary, if a bacterial CNS infection is strongly suspected but the culture results are negative, NGS may provide a definitive etiologic diagnosis. When brain abscesses occur in patients with otitis media and mastoiditis, immune-deficient patients, or trauma patients, bacteriaceae infection should not be ignored in addition to the common streptococci infection. If clinical doctors make a diagnosis of *K pneumoniae* CNS infection, they should consider the characteristics of migration infection and determine whether other metastatic septic abscesses exist. Early, regular, and long-term antibiotic therapy is important for favorable clinical outcomes.

## Author contributions

Tiantian Huo, Xintong Luo, Fan Lu, Shuo Hui.

**Conceptualization:** Jingru Zhao.

**Supervision:** Baoming Yang.

**Writing – original draft:** Jingru Zhao.

**Writing – review & editing:** Jingru Zhao, Baoming Yang.
